# A prospective examination of online social network dynamics and smoking cessation

**DOI:** 10.1371/journal.pone.0183655

**Published:** 2017-08-23

**Authors:** Amanda L. Graham, Kang Zhao, George D. Papandonatos, Bahar Erar, Xi Wang, Michael S. Amato, Sarah Cha, Amy M. Cohn, Jennifer L. Pearson

**Affiliations:** 1 Schroeder Institute for Tobacco Research and Policy Studies, Truth Initiative, Washington, District of Columbia, United States of America; 2 Department of Oncology, Georgetown University Medical Center / Cancer Prevention and Control Program, Lombardi Comprehensive Cancer Center, Washington, District of Columbia, United States of America; 3 Tippie College of Business, The University of Iowa, Iowa City, Iowa, United States of America; 4 Center for Statistical Sciences, Brown University, Providence, Rhode Island, United States of America; 5 Department of Health, Behavior and Society, The Johns Hopkins Bloomberg School of Public Health, Baltimore, Maryland, United States of America; Universite Toulouse 1 Capitole, FRANCE

## Abstract

**Introduction:**

Use of online social networks for smoking cessation has been associated with abstinence. Little is known about the mechanisms through which the formation of social ties in an online network may influence smoking behavior. Using dynamic social network analysis, we investigated how temporal changes of an individual’s number of social network ties are prospectively related to abstinence in an online social network for cessation. In a network where quitting is normative and is the focus of communications among members, we predicted that an increasing number of ties would be positively associated with abstinence.

**Method:**

Participants were N = 2,657 adult smokers recruited to a randomized cessation treatment trial following enrollment on BecomeAnEX.org, a longstanding Internet cessation program with a large and mature online social network. At 3-months post-randomization, 30-day point prevalence abstinence was assessed and website engagement metrics were extracted. The social network was constructed with clickstream data to capture the flow of information among members. Two network centrality metrics were calculated at weekly intervals over 3 months: 1) in-degree, defined as the number of members whose posts a participant read; and 2) out-degree-aware, defined as the number of members who read a participant’s post and commented, which was subsequently viewed by the participant. Three groups of users were identified based on social network engagement patterns: non-users (N = 1,362), passive users (N = 812), and active users (N = 483). Logistic regression modeled 3-month abstinence by group as a function of baseline variables, website utilization, and network centrality metrics.

**Results:**

Abstinence rates varied by group (non-users = 7.7%, passive users = 10.7%, active users = 20.7%). Significant baseline predictors of abstinence were age, nicotine dependence, confidence to quit, and smoking temptations in social situations among passive users (*p*s < .05); age and confidence to quit among active users. Among centrality metrics, positive associations with abstinence were observed for in-degree increases from Week 2 to Week 12 among passive and active users, and for out-degree-aware increases from Week 2 to Week 12 among active users (*p*s < .05).

**Conclusions:**

This study is the first to demonstrate that increased tie formation among members of an online social network for smoking cessation is prospectively associated with abstinence. It also highlights the value of using individuals’ activities in online social networks to predict their offline health behaviors.

## Introduction

Decades of research have demonstrated the importance of social ties in tobacco use and cessation [1–3, 4]. High levels of social support have been linked to better cessation outcomes [3, 5, 6] and low levels of support have been shown to be a barrier to abstinence [7, 8]. These robust associations led to numerous interventions that attempted to promote abstinence by changing the availability of support, mostly at the dyadic or small group level [9–16]. However, the effectiveness of these approaches has been mixed [17–20], leaving tobacco control researchers uncertain as to whether and how social support should be provided during the cessation process.

There are several possible explanations as to why social support interventions have been largely ineffective in promoting cessation [21]. First, it may be that social support simply cannot be manufactured or taught. Early correlational studies found that *natural* social networks were protective, whereas intervention studies have generally attempted to *manipulate* support by providing training or convening groups [22]. Second, it is also possible that dyadic or small-group interventions of current smokers quitting together did not provide sufficient diversity to impact cessation. Exposure to a more heterogeneous mix of both current and former smokers may be a more powerful influence on smoking behavior. While current smokers can provide a shared experience and empathy, former smokers can share success strategies, model abstinence-promoting behaviors, and influence norms about the acceptability of smoking. Third, it may be that previous interventions have lacked the necessary scale. Individuals are more likely to adopt a new behavior with increasing exposure to that behavior among other members of their social network [23]. In the diffusion of innovations literature this has been described as a threshold effect, or the number of contacts that an individual must have before making a decision to adopt a new process [24]. These threshold effects have been referred to as complex contagion [25], where individuals become more likely to adopt a new behavior with increasing exposure to it from other members of their social network.

With widespread Internet use [26] and the proliferation of online social networking [27], it is now feasible for current and former smokers to share information and support with thousands of others. Online social networks provide an exciting opportunity to revisit the mechanisms through which the formation and evolution of social ties may influence smoking behavior [20, 28, 29]. Information technologies enable and record asynchronous and distributed online social interactions, allowing for the use of social computing approaches to analyze an entire social network and subnetworks into which a user is embedded [30], and to identify the ties that are formed with other members over time (i.e., structural dynamics of the social network). Evaluating structural dynamics in social networks can improve network growth prediction [31], more accurately identify central network members [32, 33] and sub-communities [34], and better discriminate functional categories of connected user groups within networks [35]. Analysis of the rich data available in an online social network for smoking cessation may also yield important insights about the mechanisms through which online social ties impact offline behavior.

One such mechanism relates to the flow of information in an online network. The sharing of information–and exposure to information–is at the crux of interpersonal influence [36]. For members of an online social network to exert influence on others and ostensibly to effect change in another person’s smoking behavior requires–at a minimum–the existence of ties between and among members. One might expect that as a smoker establishes ties in a social network for smoking cessation that the exposure to cessation-promoting social influence, information, and support will increase. Numerous studies have explored online social networks across a variety of health conditions [37–43], and two recent systematic reviews and meta-analysis found that online social networks exert a positive effect on health behavior change [44, 45]. To date, studies of online networks specifically for cessation have primarily focused on describing engagement patterns [46], identifying content themes [47–54] and sentiment [55] in posts, and characterizing key network members who act as leaders [48, 50, 56, 57]. These studies provide an important foundation for understanding the impact of online social networks on smoking behavior. However, the absence of rigorous measures of smoking outcomes and the cross-sectional nature of these analyses are noteworthy shortcomings. Two recent reports [58, 59] used longitudinal data from a randomized trial to examine the prospective link between short-term online community engagement and smoking outcomes. Both reports support the causal impact of online community engagement on abstinence using individual-level utilization metrics, but neither considered this relationship from a network perspective.

This study examined online social network dynamics as predictors of smoking abstinence. Our analyses leveraged a unique dataset that blended longitudinal data from an online social network for cessation with smoking outcome data gathered on network members participating in a randomized trial. The dataset enabled us to reconstruct a large-scale online social network and to track participants’ social ties over time. We conducted a prospective exploration of abstinence using a rich set of predictors that included baseline demographic, psychosocial and smoking characteristics, website utilization metrics, and dynamic network measures. Our hypothesis was that increases in social network ties over time would signal greater exposure to cessation-related information, norms, and support with the network, and that such increases would be predictive of subsequent abstinence.

## Materials and methods

### Setting

The study was conducted within BecomeAnEX.org, a publicly available Internet cessation program. Launched in 2008, the site was developed in collaboration with the Mayo Clinic Nicotine Dependence Center [60] in accordance with national treatment guidelines [61]. A national mass media campaign [60, 62] and ongoing online advertising have resulted in over 800,000 registrants since its inception. To register on BecomeAnEX, individuals must agree to the site’s Terms of Use and Privacy Policy. The Privacy Policy states that 1) BecomeAnEX collects information about users and their use of the site; 2) Information is used for research and quality improvement purposes only; and 3) Personal information is kept confidential. Thus, de-identified data from all registered users was available for analysis. BecomeAnEX teaches problem-solving and coping skills to quit smoking, educates users about cessation medications, and facilitates social support through a large online social network. The social network is comprised of thousands of current and former smokers who interact via several asynchronous communication channels (e.g., blogs, group discussions, private messages; [30]). All user actions are date- and time-stamped and stored in a relational database.

### Participants

Participants were current smokers enrolled in a randomized trial conducted between March 2012 and January 2015 within BecomeAnEX (ClinicalTrials.gov NCT01544153). The study protocol for the randomized trial was reviewed and approved by Western Institutional Review Board (protocol #20110877). The trial protocol [63], characteristics of the trial sample [64], and the impact of the intervention arms in increasing treatment utilization [65] have been published elsewhere. Briefly, new members of BecomeAnEX were recruited to test the individual and combined effects of two potentially complementary strategies to improve cessation treatment adherence: a social network integration approach (SN) designed to integrate study participants into the BecomeAnEX social network, and access to an initial course of free nicotine replacement therapy (NRT). The study used a 2×2 randomized design to compare the effectiveness of these strategies against a web-based control (WEB). A total of 5,290 participants were randomized to WEB, WEB+SN, WEB+NRT, or WEB+SN+NRT. Smoking outcomes were gathered at 3- and 9-months post-randomization. Individuals lost to follow-up were counted as smokers.

To isolate the effects of social network dynamics on abstinence, these analyses specifically focus on the N = 2,657 trial participants in the two treatment arms that did not receive nicotine replacement therapy (WEB, WEB+SN). Participants in both conditions had full access to the BecomeAnEX website, which included the social network analyzed in this study; however, only participants in the WEB+SN arm received additional encouragement to participate in the network. Given that this is the first study to examine prospectively whether social network metrics are related to abstinence, our analyses focus on 3-month outcomes since this is the period of time when the majority of users are most likely to be active in the network [66, 67]. BecomeAnEX members who were involved in the delivery of the SN intervention were excluded from network metric calculations. Bots and spam accounts were also excluded from network calculations.

### Sources of data and measures

Analyses draw on the following sources of data: 1) baseline survey data collected during trial enrollment; 2) follow-up survey data collected at 3-months; 3) individual-level website usage metrics extracted at 3 months; and 4) social network data.

Baseline measures administered in the randomized trial assessed hypothesized moderators of treatment response and theory-driven mediators related to social network engagement [63]. Demographic variables included age, gender, employment, education, marital status, and race/ethnicity. Smoking variables included motivation to quit, the Fagerström Test for Nicotine Dependence [68], smoking rate, number of past year quit attempts, desire to quit and confidence in quitting (each on 5-point Likert scale, 1 = not at all, 5 = very much), past year use of cessation aids, advice to quit from a healthcare provider (yes/no), and whether they had ever had an illness caused or made worse by smoking (yes/no). Internet use variables included the nature and frequency of social media use. Psychosocial variables were cessation-related social support as measured by a modified version of the Partner Interaction Questionnaire [69, 70]; a subset of personality traits measured by the Ten-Item Personality Inventory (neurosis: anxious, easily upset; extraversion: extraverted, enthusiastic; openness to experience: open to new experiences, complex) [71]; and smoking temptations as measured by the nine-item short form of the Smoking Temptations Questionnaire–Short Form [72]. Two items assessed behavioral intentions (“Over the next 3 months, how likely is it that you will… 1) use BecomeAnEX regularly (i.e., at least a few times a week), 2) use nicotine replacement therapy (NRT) like the patch or gum”).

Follow-up data were gathered at 3 months via online survey and by telephone for online non-responders. Smoking abstinence was assessed using a standard measure of 30-day point prevalence abstinence (“Have you smoked cigarettes, even a puff, in the past 30 days?”).

To identify patterns of social network use, we examined each user’s behavior on two key engagement metrics: 1) pages viewed, and 2) total number of posts published. These metrics reflect a user’s overall volume of reading and online posting behaviors. Every social network page viewed by a participant was recorded, and page views were grouped into sessions, with session duration defined as the time elapsed between the first page view and the last page view in a given session. If a user did not view a new page for more than 30 minutes, the system marked them as inactive and their next return visit created a new session. Total number of posts is a included original content and replies across communication channels.

General utilization metrics were extracted at 3 months and included number of return visits, minutes spent using the site, and number of days logged in to the site. Social network engagement metrics were divided into measures of passive (reading) and active (posting) engagement. Passive engagement metrics included counts of profiles viewed, blog posts read, and private messages received. Active engagement metrics included counts of blog posts/comments, group discussion posts, wall posts, and private messages sent.

### Social network metrics and analyses

The Python programming package NetworkX (v. 1.11) was used to construct and analyze the BecomeAnEX social network. The network spanned January 2010 through May 2015. In-degree and out-degree centrality metrics were calculated based on tie formation during each of the 12 weeks in the 3-month study period. Ties were cumulative, so that the network constructed for each week included all ties created during that week, as well as all ties created during earlier weeks. As previously described [30], we used URL clickstream data to determine the formation of a tie, based on an interaction in the network through active (posting) and/or passive (reading) behavior. Each node represents an individual user. A directed tie pointing from Mary to John means that John accessed content written by Mary.

In a directed network, a node’s *in-degree* refers to the number of other nodes that have ties pointing to it (i.e., the number of people who may have influenced that user). Those who have read posts written by many others will have high in-degree. A node’s *out-degree* is the number of its outgoing ties (i.e., the number of people a user has potentially influenced), which increases when another member reads a post they have authored. Out-degree is a useful metric for examining an individual’s influence on or importance to others in the network. However, because out-degree is inherently unidirectional, it is of less utility for predicting a member’s own behavioral outcome. A user can continue to accumulate out-degree long after they have disengaged from an online network and/or from the process of quitting entirely. In addition, out-degree does not account for the fact that a person's behavior both influences and is influenced by their environment, the notion of reciprocal determinism as described by Bandura [73]. If a user is unaware of how many (or few) others they have influenced, there is little reason to expect that posting content alone would be sufficient to exert an impact on their likelihood of abstinence. To address this issue, we introduce a novel metric, *out-degree-aware*, which measures only the number of people a member is aware that he/she has influenced. Out-degree-aware was operationalized similar to traditional out-degree, but was restricted to only count outgoing ties (e.g., from Mary to John) when (1) John subsequently responded or commented in the same thread where he read Mary’s original content, and (2) clickstream data indicated that Mary had viewed John’s response. Given our focus on individual-level behavior change in the context of a social network, we use out-degree-aware instead of simple out-degree in all of our analyses.

By incorporating both posting and reading behaviors, our analyses capture how information flows among users via each communication channel. Network metrics were aggregated for social network ties across blogs, group discussions, and private messages. The fourth communication channel, message boards, was not included because the site architecture did not allow clickstream data to be established as unambiguously as it could be for the other three channels. We identified the largest strongly connected component (LSCC), defined as the largest subnetwork in which there is a directed path between every pair of participants. We report average path length, defined as the average number of steps along the shortest paths for all possible pairs of network nodes in the LSCC. It is a measure of the efficiency of information spread in a network. We also calculated clustering coefficient, which measures the probability of triads in a social network and reflects cohesion within the network. Finally, we calculated individuals’ centralities at weekly intervals over the first 3 months of the trial.

### Statistical analyses

Based on two key social network utilization metrics (both dichotomized at zero), we were able to divide study participants into three distinct groups: a) non-users, with no posts or page views; b) passive users, with positive page views, but no posts; and c) active users, with both page views and posts. We then characterized these groups on distinguishing baseline characteristics and 3-month website utilization and social network metrics. For between-group comparisons of baseline characteristics, one-way analysis of variance and chi-square tests were used for continuous and categorical variables, respectively. Due to the skewness of continuous website utilization and network metrics, and the small group sizes in categorical metrics, Kruskal-Wallis rank sum tests and Fisher's exact tests (as implemented in R [74] for 2x3 tables [75, 76] were used for continuous and categorical variables, respectively.

Next, stepwise logistic regression with alpha = .10 as the significance threshold was used to choose group-specific reference models from all available baseline covariates and website utilization measures. Once reference models were established for each group, we added network centrality metrics to each model to test our primary hypothesis that an increasing number of social ties over time would be positively associated with abstinence, controlling for baseline characteristics and website utilization. Previous studies have shown that website engagement [77] and network tie formation [78] tend to be greatest during a user’s first week, and that early experiences in an online social network are the most critical to continued engagement [79]. Based on these findings, we examined the links between each user’s centrality at the end of the first week with abstinence, as well as the change in centrality over the remaining study period through 12 weeks. Thus, the following four network metrics were added to our models: 1) in-degree ties formed during the first week; 2) out-degree-aware ties formed during the first week; 3) in-degree ties formed during weeks 2 to 12; and 4) out-degree-aware ties formed during weeks 2 to 12. All network centrality metrics were analyzed in the square root scale, but were otherwise left unstandardized.

Given that passive users had zero out-degrees by definition, we considered an in-degree model alone for this group, whereas we fit a more elaborate model for active users that included both in-degree and out-degree-aware centrality metrics. To enhance the interpretability and comparability of the remaining model parameters, continuous baseline and website utilization predictors were centered by the cluster median and scaled by the distance from the median to the third cluster quartile. As a result, their regression coefficients capture the change in the log-odds of abstinence for a one-quartile increase in the predictor above its cluster median. Between-subject correlation in abstinence outcomes was accommodated via a Generalized Estimating Equations (GEE) approach, with a working independence correlation matrix used to robustify model-based standard error estimates. Model predictiveness was assessed using Area Under the Curve (AUC), with ten-fold cross-validation used to correct for over-optimism due to the use of the same dataset for both training and validation purposes. The study protocol for these analyses was approved by Chesapeake IRB (protocol #00010302).

## Results

### Social network structure

The social network consisted of 16,812 nodes with at least one tie. There were more than 705,224 ties, each of which was date- and time-stamped based on when the tie was formed. Of the 16,812 nodes, 11,112 participants (66%) were identified as passive users who read others’ content but did not post. Of the remaining 5,700 participants who posted content, 5,315 (31.6% of all nodes) could reach each other via directed paths and hence constitute the LSCC. Within the LSCC, the average path length between any pair of nodes is 2.25 hops, reflecting a well-connected network among members that had ever posted. The clustering coefficient of the network is 0.766, which is higher than many other social networks (78). This suggests a cohesive social network, in which two neighbors of the same node are connected with a probability of 76.6%.

**Fig 1:**
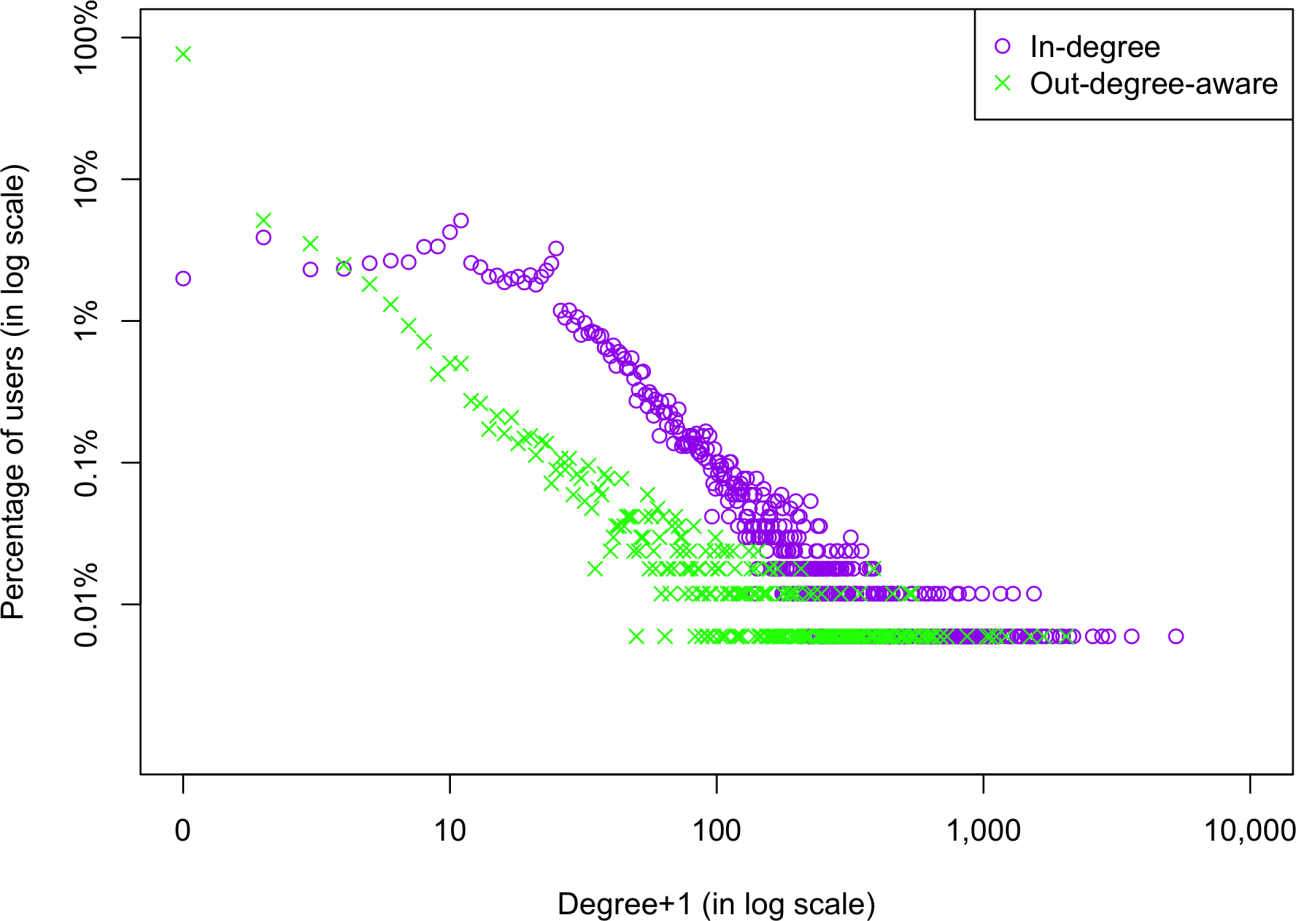
Distributions of Week 1 degrees for all users in the network.

**Fig 2:**
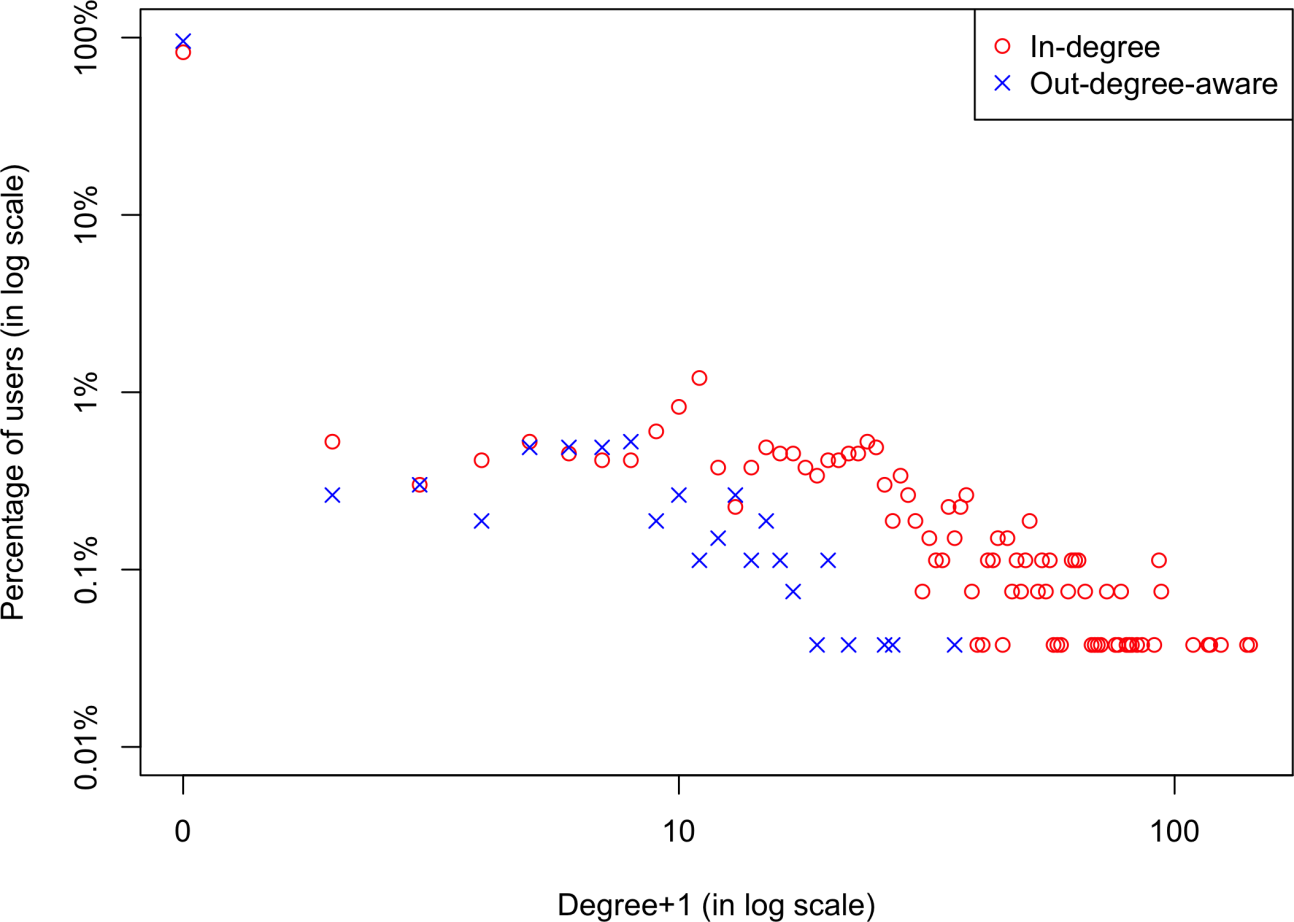
Distributions of Week 1 degrees for the 2,657 users in the analytic sample.

The distributions of Week 1 in-degrees and out-degrees are shown for all users in the network (Fig 1), and for the 2,657 users in the analytic sample (Fig 2). Both figures feature highly skewed degree distributions: most users had low degrees, but a small number had very high degrees. In Fig 1, 7.4% of all users had in-degree over 100, with the maximum in-degree being 5,268; 1.4% had out-degree-aware over 100, with the maximum out-degree being 2,041. As shown in Fig 2, study participants had lower degrees compared to all users in the network: 11.7% had in-degree over 10, with the maximum in-degree of 141; 1.2% had out-degree-aware over 10, with the maximum out-degree-aware of 35. These findings are to be expected given that the degree distributions in Fig 1 aggregate all users’ degrees over years, while Fig 2 only reflects Week 1 ties of the 2,657 users. Moreover, distribution curves for in-degrees are generally above those for out-degrees-aware, because it is easier to accumulate in-degrees (by reading others’ contributions) than out-degrees-aware (by posting and attracting readers, and revisiting).

### Sample description by social network engagement patterns

Three groups of users were identified based on social network utilization patterns: non-users (N = 1,362), passive users (N = 812), and active users (N = 483). As shown in Table 1, intensity of social network engagement was positively correlated with abstinence (no smoking within the past 30 days): active users had the highest abstinence rate (20.7%), followed by passive users (10.7%), and non-users (7.7%).

**Table 1:** Social network utilization patterns and smoking outcomes among 3 utilization groups.

Group	N	Total number of posts^a^; Median (IQR)	Social network page views; Median (IQR)	30-day PPA at 3 months; n (%)
Non-users	1362	0 (0–0)	0 (0–0)	105 (7.7)
Passive users	812	0 (0–0)	5 (2–10)	87 (10.7)
Active users	483	2 (1–6.5)	34 (15–87.5)	100 (20.7)

^a^ Posts–messages created by users in blogs, group discussions, message board; does not include private messages

IQR: interquartile range

PPA: point prevalence abstinence, counting all survey non-responders as smokers

Additional group-specific summaries (Table 2) suggest the existence of positive relationships between social network engagement and older age, some college education, lack of full-time employment, prior illness from smoking, and intention to use BecomeAnEX regularly. Passive users were more likely to be non-Hispanic and to score lower on extraversion. Female gender, a higher level of smoking temptations in negative affect situations, having a profile on one or more social networking sites, and prior use of behavioral quit methods were key identifying characteristics of active users.

**Table 2:** Baseline characteristics by group.

	Analytic Sample (N = 2,657)^a^	Non-Users (N = 1,362)	Passive Users (N = 812)	Active Users (N = 483)	P-value^b^
**Demographic Variables**					
Female, N (%)	1630 (61.3)	802 (58.9)	490 (60.3)	337 (69.8)	< .001
Age, years, M (SD)	42.1 (13.2)	41 (13.3)	42.9 (13.3)	44 (12.6)	< .001
Marital status (partner: yes), N (%)	977 (36.8)	481 (35.3)	299 (36.8)	197 (40.8)	.101
Race					
Black or African American, N (%)	409 (15.4)	222 (16.3)	115 (14.2)	72 (14.9)	.388
White, N (%)	2130 (80.2)	1080 (79.1)	663 (81.7)	390 (80.7)	.325
Other, N (%)	118 (4.4)	63 (4.6)	34 (4.2)	21 (4.4)	.886
Ethnicity, Hispanic, N (%)	162 (6.1)	99 (7.3)	33 (4.1)	30 (6.2)	.010
Education: Some college or more, N (%)	1940 (72.9)	968 (71.1)	594 (73.2)	376 (77.8)	.016
Employed full-time, N (%)	1210 (45.5)	658 (48.3)	363 (44.7)	189 (39.1)	.002
**Smoking Variables**					
Readiness to quit: next 30 days, N (%)	2210 (83.1)	1110 (81.6)	690 (85.0)	405 (83.9)	.118
Smoking frequency, daily, N (%)	2560 (96.3)	1310 (96.5)	778 (95.8)	466 (96.5)	.707
Time to first cigarette					
5 min or less, N (%)	1010 (38.1)	535 (39.3)	297 (36.6)	181 (37.5)	.431
6–30 min, N (%)	1080 (40.7)	542 (39.8)	330 (40.6)	209 (43.3)	.409
31 min or more, N (%)	563 (21.2)	285 (20.9)	185 (22.8)	93 (19.3)	.305
Fagerström score, M (SD)	5.21 (2.2)	5.21 (2.3)	5.18 (2.3)	5.26 (2.1)	.851
Cigarettes per day, M (SD)	16.5 (8.4)	16.4 (8.6)	16.8 (8.2)	16.3 (7.8)	.432
Quit attempts in past year, M (SD)	3.18 (12.6)	2.82 (7.4)	3.77 (19.8)	3.18 (7.6)	.236
Desire to quit, M (SD)	4.55 (0.6)	4.54 (0.6)	4.54 (0.6)	4.61 (0.6)	.090
Confidence to quit, M (SD)	3.3 (1.1)	3.29 (1.1)	3.27 (1.1)	3.39 (1.0)	.129
Quit methods					
# Behavioral methods used, M (SD)	0.2 (0.5)	0.2 (0.5)	0.2 (0.5)	0.3 (0.6)	.032
# Medicines used, M (SD)	0.6 (0.9)	0.5 (0.9)	0.6 (1.0)	0.6 (1.0)	.103
# Alternative methods used, M (SD)	0.6 (0.8)	0.6 (0.8)	0.6 (0.9)	0.6 (0.8)	.792
**Health Status**					
Illness from smoking, yes, N (%)	1690 (63.6)	826 (60.6)	532 (65.5)	331 (68.5)	.003
Doctor advice to quit, yes, N (%)	1660 (62.3)	838 (61.5)	508 (62.6)	310 (64.2)	.578
**Smoking temptations**					
Social subscale, M (SD)	4.0 (0.8)	4.1 (0.8)	4.0 (0.8)	4.0 (0.8)	.126
Negative affect subscale, M (SD)	4.5 (0.6)	4.5 (0.7)	4.5 (0.7)	4.6 (0.6)	.005
Craving subscale, M (SD)	3.8 (0.8)	3.8 (0.8)	3.8 (0.8)	3.8 (0.8)	.407
**Cessation related social support**					
PIQ—positive subscale, M (SD)	6.3 (5.0)	6.3 (5.0)	6.3 (5.0)	6.0 (4.9)	.344
PIQ—negative subscale, M (SD)	4.4 (4.6)	4.6 (4.7)	4.4 (4.6)	4.1 (4.4)	.202
**Personality traits**					
Extraversion, M (SD)	8.6 (3.2)	8.7 (3.1)	8.3 (3.3)	8.7 (3.3)	.024
Neurosis, M (SD)	8.3 (3.1)	8.4 (3.1)	8.3 (3.1)	8.3 (3.1)	.517
Openness to experience, M (SD)	10.8 (2.5)	10.8 (2.5)	10.7 (2.5)	10.9 (2.4)	.210
**Internet and social network use**					
Use Internet to communicate with others, several times a day, N (%)	1020 (38.4)	529 (38.8)	298 (36.7)	194 (40.2)	.419
Any social network profile, yes, N (%)	2390 (89.8)	1210 (89.1)	720 (88.7)	453 (93.8)	.006
Use social networking site, several times a day, N (%)	1330 (49.9)	688 (50.5)	393 (48.4)	246 (50.9)	.565
Send messages on social networking site, several times a day, N (%)	641 (24.1)	321 (23.6)	199 (24.5)	121 (25.1)	.770
**Behavioral intentions**					
Intention to use EX regularly, probably/definitely, N (%)	2600 (97.7)	1320 (97.1)	796 (98.0)	478 (99.0)	.043
Intention to use medication, probably/definitely, N (%)	1540 (57.9)	825 (60.6)	445 (54.8)	268 (55.5)	.015

^a^ Participants in WEB and WEB+SN, excluding 3 individuals with missingness in their clickstream data.

^b^ P-values calculated using chi-square tests for categorical variables and one-way ANOVA for continuous variables.

Given that the three groups were generated on the basis of network engagement metrics, it is not surprising that Table 3 shows them to be strictly ordered in terms of both passive and active engagement. The groups are also ordered in terms of website utilization levels, both general (i.e., return visits, time on site, total page views) and specific (i.e., skills training page views), suggesting a strong positive association between social network engagement and website utilization.

**Table 3:** Website utilization and social network metrics by cluster at 3 months^a^.

	Analytic Sample (N = 2,657)^b^	Non-Users (N = 1,362)	Passive Users (N = 812)	Active Users (N = 483)	P-val^c^
**Website Utilization Metrics**	** **	** **	** **	** **	
Return visits, Mdn (IQR)	2 (1–4)	1 (1–2)	3 (2–5)	6 (3–13)	< .001
Time on site, minutes, Mdn (IQR)	18 (4–45)	5 (0–18)	29 (14–51)	87 (47–189)	< .001
Skills training page views, Mdn (IQR)	16 (5–35)	7 (2–19)	24 (11–42)	43 (24–75)	< .001
Passive social network engagement					
Viewed 1+ profiles, N (%)	656 (24.7)	0 (0)	350 (43.1)	306 (63.4)	< .001
Read 1+ blog posts, N (%)	355 (13.4)	0 (0)	140 (17.2)	215 (44.5)	< .001
Received 1+ private messages, N (%)	605 (22.8)	230 (16.9)	188 (23.2)	187 (38.7)	< .001
Active social network engagement					
Wrote 1+ blog posts, N (%)	202 (7.6)	0 (0)	1 (0.123)	201 (41.6)	< .001
Wrote 1+ blog comments, N (%)	191 (7.19)	0 (0)	0 (0)	191 (39.5)	< .001
Wrote 1+ group discussion posts, N (%)	36 (1.35)	0 (0)	9 (1.11)	27 (5.59)	< .001
Wrote 1+ wall posts, N (%)	275 (10.4)	0 (0)	3 (0.369)	272 (56.3)	< .001
Sent 1+ private messages, N (%)	122 (4.59)	0 (0)	1 (0.123)	121 (25.1)	< .001
**Social Network Centrality Metrics**					
In-degree, Week 1, Mdn (IQR)	0 (0–0)	0 (0–0)	0 (0–0)	5 (0–23)	< .001
In-degree change, Weeks 2–12, Mdn (IQR)	0 (0–0)	0 (0–0)	0 (0–0)	0 (0–12)	< .001
Out-degree-aware, Week 1, Mdn (IQR)	0 (0–0)	0 (0–0)	0 (0–0)	0 (0–0)	< .001
Out-degree-aware change, Weeks 2–12, Mdn (IQR)	0 (0–0)	0 (0–0)	0 (0–0)	0 (0–0)	< .001

^a^ 3-month metrics presented unless otherwise specified

^b^ Excludes 3 individuals due to missingness in their clickstream data

^c^ P-values calculated using Fisher's exact test for categorical variables and Kruskal-Wallis rank sum test for continuous variables (due to skewnewss).

IQR: interquartile range

### Social network dynamics and abstinence outcomes

We present logistic regression models for the two user groups with positive social network ties: one for the N = 812 passive users and another for the N = 483 active users.

Table 4 includes all baseline characteristics that showed at least a trend towards significance (p < .10) among passive users, before network metrics were added to the regression model. Controlling for these baseline characteristics, Week 1 in-degree showed no relationship with abstinence (p = .78). However, in-degree tie formation during Weeks 2–12 was statistically significant (p = .04), with the odds of abstinence higher by roughly 20% among users who accumulated one additional tie after the first week following website registration (OR = 1.19, 95% CI = 1.00–1.41), and two thirds higher among users who accumulated nine such ties (OR = 1.68, 95% CI = 1.00–2.80, data not shown).

**Table 4:** Odds Ratio (OR) estimates of 3 month abstinence from the GEE logistic regression model for passive participants (N = 812).

Variable category	Variable name	OR	LCL	UCL	P-val
*Intercept*	(Intercept)	0.09	0.05	0.16	< .001
*Baseline*	Age^a^	0.78	0.66	0.92	.004
	Education: Some College or more	1.58	0.89	2.80	.121
	Time to first cigarette: 5 min or less	0.40	0.22	0.71	.002
	Confidence to quit^a^	1.27	1.02	1.59	.033
	Smoking temptations, social subscale^a^	1.25	1.01	1.56	.045
	Smoking temptations, negative affect subscale^a^	0.90	0.80	1.01	.075
	Smoking temptations, craving subscale^a^	1.09	0.97	1.23	.142
	Extraversion^a^	0.83	0.68	1.01	.062
*Network*	In-degree, Week 1^b^	1.02	0.90	1.15	.781
	In-degree change, Weeks 2 to 12^b^	1.19	1.00	1.41	.044

^a^ Standardized using Location = Median, Scale = 3^rd^ Quartertile–Median.

Median (Inter-Quartile Range): Age = 45 (32–53); Confidence to quit = 3 (3–4); Extraversion = 8 (6–11); Smoking temptations: social = 4 (3.7–4.7); negative affect = 4.7 (4–5); craving = 4 (3.3–4.3).

^b^ Both initial value and change transformed to the square root scale.

GEE: Generalized Estimating Equations; UCL/LCL: 95% Upper & Lower Confidence Limits.

Additional variables associated with abstinence among passive users at the 5% level of significance were: older age (53 vs. 45 years: OR = .78, 95% CI = .66-.92), smoking within 5 minutes of waking (OR = .40, 95% CI = .22-.71), unit increases in confidence to quit (OR = 1.27, 95% CI = 1.02–1.59), and temptations to smoke in social situations (4.7 vs. 4.0: OR = 1.25, 95% CI = 1.01–1.56).

Table 5 includes all baseline characteristics that showed at least a trend towards significance (p < .10) among active users, before network metrics were added to the regression model. Controlling for these baseline characteristics, Week 1 in-degree and out-degree-aware ties showed no relationship with abstinence (p = .159, .83, respectively). However, additional tie formation during Weeks 2–12 ties was significant for both in-degree (p = .024) and out-degree-aware (p = .035), with the odds of abstinence among users that accumulated just one additional tie after the first week rising by 14% for in-degree ties (OR = 1.14, 95% CI = 1.02–1.28) and by 29% for out-degree-aware ties (OR = 1.29, 95% CI = 1.02–1.63). For users that accumulated nine such ties, the odds of abstinence rose by half for in-degree ties (OR = 1.48, 95% CI = 1.06–2.10) and more than doubled for out-degree ties (OR = 2.15, 95% CI = 1.06–4.33, data not shown). Additionally, older age (54 vs. 45 years: OR = .84, 95% CI = .71–1.00) and unit increases in confidence to quit (OR = 1.69, 95% CI = 1.31–2.19) were both associated with abstinence at the 5% level of significance.

**Table 5:** Odds Ratio (OR) estimates of 3 month abstinence from the gee logistic regression model for active participants (N = 483).

Variable category	Variable name	OR	LCL	UCL	P-val
*Intercept*	(Intercept)	0.06	0.03	0.14	< .001
*Baseline*	Age^a^	0.84	0.71	1.00	.045
	Race: White vs. not	1.67	0.84	3.29	.142
	Confidence to quit^a^	1.69	1.31	2.19	< .001
	Doctor advice to quit: yes	1.46	0.86	2.49	.157
	Neurosis^a^	1.21	0.99	1.47	.057
*Network*	In-degree, Week 1^b^	0.93	0.84	1.03	.159
	In-degree change, Weeks 2 to 12^b^	1.14	1.02	1.28	.024
	Out-degree-aware, Week 1^b^	1.03	0.81	1.29	.830
	Out-degree-aware change, Weeks 2 to 12^b^	1.29	1.02	1.63	.035

^a^ Standardized using Location = Median, Scale = 3^rd^ Quartile–Median.

Median (Inter-Quartile Range): Age = 45 (34–54); Confidence to quit = 3 (3–4); Neurosis = 8 (6–10.5).

^b^ Both initial value and change transformed to the square root scale.

GEE: Generalized Estimating Equations; UCL/LCL: 95% Upper & Lower Confidence Limits.

In terms of predictive ability, adding network centrality metrics to a model containing only baseline participant characteristics left the AUC unchanged at .63 in Table 4, but raised it from .65 to .73 in Table 5. To understand this discrepancy, it helps to examine the sample distribution of the network centrality metrics, as shown in S1 Table. It is seen that 91.9% of passive users accumulated no additional in-degree ties during Weeks 2–12 of the study, limiting the relevance of any beneficial effects of stronger network integration to the top 8.1% of the sample. In contrast, only 62.7% of active users accumulated no additional in-degree ties during Weeks 2–12 of the study, rising to 78.7% for out-degree-aware ties. Therefore, model findings regarding change in network centrality metrics are relevant to a much larger proportion of active than passive users.

## Discussion

To our knowledge, this is the first study to examine prospectively the association between social network dynamics and abstinence in an online social network for smoking cessation. Dynamic analysis of individuals’ network positions can provide more information about one’s engagement in a social network than mere snapshots of network configurations at the beginning or end of a trial. Across both types of network users, forming new ties during Weeks 2–12 of the study was predictive of subsequent abstinence, where initial activity during Week 1 was not. Among passive users (“lurkers”), in-degree change remained significant even after controlling for age, confidence in quitting, nicotine dependence, and smoking self-efficacy; among active users (“contributors”), change in both in-degree and out-degree-aware remained significant after controlling for age and confidence in quitting. These findings suggest that even after controlling for important baseline covariates, sustained increases in exposure to information and influence from other members of an online social network for smoking cessation are independent predictors of success in quitting smoking.

Three groups of users emerged based on intensity of social network engagement, with abstinence rates ordered by level of engagement. At 3-months after registration, 8% of non-users were abstinent, compared to 11% of passive users and 21% of active users. This research adds to previous work that has documented better outcomes with higher levels of website engagement [80–83]. While we cannot rule out the possibility that self-selection is at least partly at play with these findings, the fact that sustained increases in social network tie formation were retained as predictors in models that accounted for a broad set of baseline participant characteristics increases our confidence in the role of network exposure in causally promoting abstinence. Determining the causal impact of social network participation on health behavior change is inherently challenging, since it may not be feasible (or even prudent) to randomize participants to “use” or “not use” an online community [59]. The long history of social support interventions that have been largely unsuccessful in increasing quit rates supports the notion that interpersonal relationships meaningful enough to spur behavior change cannot be randomized. Individuals decide whether and how to participate in online networks based on their own unique needs and desires for information and support, their interest in finding “similar others,” and their ability to form interpersonal relationships, among many other reasons [84]. Dynamic social network analyses provide an alternate lens to study this phenomenon.

Our analyses also revealed that the formation of social ties over time was more predictive of cessation than ties formed during an initial period of engagement. This finding would not have been observed by alternative analyses that aggregated across time. These findings lay important groundwork for future exploration of social contagion for behavior change in online social networks. For example, more fine-grained analysis of the exposure a user received would make it possible to study the diffusion of abstinence among individuals in the online social network.

Three limitations of this work should be noted. First, these analyses examined short term abstinence to determine whether an initial signal exists for social network dynamics on abstinence. Future research should examine whether dynamic positions in a network over a longer period of time are related to sustained abstinence. Second, we measured a user’s integration into the online social network as reflected by degree centralities. While intuitive and popular, degree centralities do not capture who a user’s neighbors are (e.g., another user in the core or at the periphery of the network) or the strength of ties with the user’s network neighbors. Third, our decision to focus on a user’s network position at the end of their first week was informed by previous research, but may have been too coarse to detect more rapid effects of network position change. Future research should explore alternative approaches to operationalizing network dynamics.

Strengths of this study include the availability of abstinence measures gathered through a randomized trial in conjunction with both rich and novel social network metrics. This unique dataset allowed us to examine changes in online network position over time and their association with offline cessation outcomes. In addition, the ability to examine both in-degree and out-degree-aware allowed us to parse out which particular types of social network engagement are most critical. This work introduces a novel measure of out-degree-aware which has two noteworthy advantages: 1) from a network perspective, this is a more accurate measure of one's influence, as it captures both the sphere of influence of one's contribution/post, and the activity level of the contributor in the social network, and 2) from a psychological perspective, this measure can potentially reflect the level of self-fulfillment, sense of achievement, or perceived social support one gets from contributing to the network. These are areas worthy of further exploration. Finally, this work provides a model for blending dynamic social network analysis with traditional methods of examining outcomes in a smoking cessation trial, and elucidates the ways in which engagement in an online intervention may translate into improved abstinence rates. Importantly, this research begins to unpack the “black box” of online interventions to identify the active ingredients [85] and addresses the call for more research on the mechanisms through which the ties that are formed online translate into meaningful behavior change [29].

## Supporting information

S1 Table In-degree and out-degree-aware frequencies by social network utilization group. (DOCX)
