# Supplementary material for: A prospective examination of online social network dynamics and smoking cessation
Source: PLoS One. 2017 Aug 23;12(8):e0183655. doi: 10.1371/journal.pone.0183655 (PMC5568327; doi:10.1371/journal.pone.0183655)
Supplement: S1 Table — (DOCX) [file pone.0183655.s001.docx]

| S1 Table. In-degree and out-degree-aware frequencies by social network utilization group. | | | | | | | | | | | | | | | | | | | | |
| --- | --- | --- | --- | --- | --- | --- | --- | --- | --- | --- | --- | --- | --- | --- | --- | --- | --- | --- | --- | --- |
|  |  | **In-degree ties**  **at Week 1** | | | |  | **In-degree tie accumulation Weeks 2-12** | | | |  | **Out-degree-aware ties at Week 1** | | | |  | **Out-degree-aware tie accumulation Weeks 2-12** | | | |
|  |  | **PASSIVE** | | **ACTIVE** | |  | **PASSIVE** | | **ACTIVE** | |  | **PASSIVE** | | **ACTIVE** | |  | **PASSIVE** | | **ACTIVE** | |
|  |  | **(N=812)** | | **(N=483)** | |  | **(N=812)** | | **(N=483)** | |  | **(N=812)** | | **(N=483)** | |  | **(N=812)** | | **(N=483)** | |
| **Degree** |  | **n** | **%** | **n** | **%** |  | **n** | **%** | **n** | **%** |  | **n** | **%** | **n** | **%** |  | **n** | **%** | **n** | **%** |
| 0 |  | 618 | 76.1 | 215 | 44.5 |  | 746 | 91.9 | 303 | 62.7 |  | 812 | 100.0 | 363 | 75.2 |  | 812 | 100.0 | 380 | 78.7 |
| 1 |  | 8 | 1.0 | 6 | 1.2 |  | 11 | 1.4 | 12 | 2.5 |  | 0 | 0.0 | 7 | 1.4 |  | 0 | 0.0 | 8 | 1.7 |
| 2 |  | 3 | 0.4 | 5 | 1.0 |  | 3 | 0.4 | 5 | 1.0 |  | 0 | 0.0 | 8 | 1.7 |  | 0 | 0.0 | 8 | 1.7 |
| 3 |  | 6 | 0.7 | 5 | 1.0 |  | 1 | 0.1 | 5 | 1.0 |  | 0 | 0.0 | 5 | 1.0 |  | 0 | 0.0 | 7 | 1.4 |
| 4 |  | 4 | 0.5 | 10 | 2.1 |  | 3 | 0.4 | 7 | 1.4 |  | 0 | 0.0 | 13 | 2.7 |  | 0 | 0.0 | 12 | 2.5 |
| 5 |  | 6 | 0.7 | 6 | 1.2 |  | 4 | 0.5 | 4 | 0.8 |  | 0 | 0.0 | 13 | 2.7 |  | 0 | 0.0 | 5 | 1.0 |
| 6 |  | 4 | 0.5 | 7 | 1.4 |  | 3 | 0.4 | 4 | 0.8 |  | 0 | 0.0 | 13 | 2.7 |  | 0 | 0.0 | 4 | 0.8 |
| 7 |  | 7 | 0.9 | 4 | 0.8 |  | 3 | 0.4 | 3 | 0.6 |  | 0 | 0.0 | 14 | 2.9 |  | 0 | 0.0 | 5 | 1.0 |
| 8 |  | 7 | 0.9 | 9 | 1.9 |  | 2 | 0.2 | 5 | 1.0 |  | 0 | 0.0 | 5 | 1.0 |  | 0 | 0.0 | 3 | 0.6 |
| 9 |  | 12 | 1.5 | 10 | 2.1 |  | 3 | 0.4 | 5 | 1.0 |  | 0 | 0.0 | 7 | 1.4 |  | 0 | 0.0 | 5 | 1.0 |
| 10+ |  | 137 | 16.9 | 206 | 42.7 |  | 33 | 4.1 | 130 | 26.9 |  | 0 | 0.0 | 35 | 7.2 |  | 0 | 0.0 | 46 | 9.5 |
| Total |  | 812 | 100.0 | 483 | 100.0 |  | 812 | 100.0 | 483 | 100.0 |  | 812 | 100.0 | 483 | 100.0 |  | 812 | 100.0 | 483 | 100.0 |
